# Supplementary material for: Dietary Probiotics Modulate Gut Barrier and Immune-Related Gene Expression and Histomorphology in Broiler Chickens under Non- and Pathogen-Challenged Conditions: A Meta-Analysis
Source: Animals (Basel). 2023 Jun 13;13(12):1970. doi: 10.3390/ani13121970 (PMC10295439; doi:10.3390/ani13121970)
Supplement: Supplementary file 1 [file animals-13-01970-s001.zip › animals-2415174-supplementary.pdf]

animals

MDPI

Article

Dietary Probiotics Modulate Gut Barrier and Immune-Related Gene Expression and Histomorphology in Broiler Chickens under Non- and Pathogen-Challenged Conditions: A Meta-Analysis

Fitra Yosi [1,2,3] and Barbara U. Metzler-Zebeli [1,2,*]

[1] Unit Nutritional Physiology, Institute of Physiology, Pathophysiology, and Biophysics, Department of Biomedical Sciences, University of Veterinary Medicine Vienna, 1210 Vienna, Austria; fitra.yosi@vetmeduni.ac.at or fitrayosi@unsri.ac.id
[2] Christian-Doppler Laboratory for Innovative Gut Health Concepts of Livestock, University of Veterinary Medicine Vienna, 1210 Vienna, Austria
[3] Department of Animal Science, Faculty of Agriculture, University of Sriwijaya, Palembang 30662, Indonesia
* Correspondence: barbara.metzler@vetmeduni.ac.at

Citation: Yosi, F.; Metzler-Zebeli, B.U. Dietary Probiotics Modulate Gut Barrier and Immune-Related Gene Expression and Histomorphology in Broiler Chickens under Non- and Pathogen-Challenged Conditions: A Meta-Analysis. *Animals* **2023**, *13*, 1970. https://doi.org/10.3390/ani13121970

Academic Editor: Vincenzo Tufarelli

Received: 9 May 2023
Revised: 1 June 2023
Accepted: 9 June 2023
Published: 13 June 2023

Copyright: © 2023 by the authors. Licensee MDPI, Basel, Switzerland. This article is an open access article distributed under the terms and conditions of the Creative Commons Attribution (CC BY) license (https://creativecommons.org/licenses/by/4.0/).

Table S1. List of references and the respective experimental variables included in the meta-analysis.

				Pathogen				Gut Segments				Variables			
**Study**	**Reference**	**Basal Diet[3]**	**Dietary Probiotics[1]**	**No**	**Yes**	**Types[2]**	**Route**	**D**	**J**	**I**	**C**	**BAR**	**IMU**	**HIS**	**GRO**
1	Zhen et al. (2018)	corn, soybean meal	*B. coagulans*	x	x	*S. enteritidis*	oral		x			x	x	x	x
2	Kan et al. (2021)	corn, wheat, soybean meal	*B. licheniformis*		x	*E. maxima, C. perfringens*	oral		x			x	x		x
3	Zhang et al. (2021)	corn, soybean meal, fish meal	*B. coagulans*	x						x		x			x
4	Li et al. (2015)	corn, soybean meal, corn gluten meal	*B. amyloliquefaciens*		x	*Escherichia coli*	injection		x	x		x	x	x	x
5	Liu et al. (2021)	corn, soybean meal	*B. subtilis*		x	*C. perfringens*	oral		x			x	x		
6	Wang et al. (2021)	corn, soybean meal, corn gluten meal	*B. subtilis*		x	*C. perfringens*	oral		x			x		x	x
7	Wu et al. (2018)	corn, soybean meal	*B. coagulans*	x	x	*E. maxima, E. tenella, C. perfringens*	oral		x			x	x	x	x
8	Musa et al. (2019)	corn, wheat, distiller grain, soybean meal, corn protein powder, peanut meal,	*B. subtilis, B. licheniformis*		x	*C. perfringens*	oral	x	x	x		x		x	x
9	Bilal et al. (2021)	corn, soybean meal	*B. subtilis, B. pumilus*	x						x		x			x
10	Chaudhari et al. (2020)	corn, soybean meal	*B. licheniformis, B. amyloliquefaciens*		x	*E. maxima, E. tenella, E. acervulina*	oral		x		x	x	x		
11	Wu et al. (2021)	corn, soybean meal	*L. acidophilus*	x	x	*Escherichia coli*	oral		x	x		x	x	x	x
12	Xu et al. (2021)	corn, bran, soybean meal, cottonseed	*C. butyricum*	x	x	*E. maxima, C. perfringens*	oral			x		x	x		

		meal, corn gluten meal												
13	Wu et al. (2019)	corn, soybean meal	*E. faecium*	x	x	*C. perfringens*	oral	x			x	x		
14	He et al. (2019)	corn, soybean meal, fish meal, corn gluten meal	*B. subtilis, B. licheniformis, S. cerevisiae*	x				x	x		x		x	x
15	Memon et al. (2021)	corn, soybean meal	*B. subtilis*		x	*E. tenella*	oral			x	x			
16	Mountzouris et al. (2019)	corn, soybean meal	*L. reuteri, L. salivarius, E. faecium, Bi. animalis, P. acidilactici*	x					x		x			
17	Wu et al. (2019)	corn, wheat, soybean meal, fish meal	*L. plantarum, Pa. polymyxa*	x				x	x		x			
18	Huang et al. (2019)	corn, wheat, soybean meal	*E. faecium*		x	*Escherichia coli*	injection	x			x	x	x	
19	Bai et al. (2013)	corn, soybean meal, rapeseed meal, cottonseed meal	*L. fermentum, S. cerevisiae*	x				x				x		x
20	Yang et al (2021)	corn, bran, soybean meal, fish meal	*B. subtilis*		x	*E. tenella*	oral			x	x			
21	Wang et al. (2016)	corn, soybean meal	*S. cerevisiae*	x	x	*Escherichia coli*	oral	x	x			x	x	x
22	Chang et al. (2020)	corn, soybean meal	*L. acidophilus, L. fermentum, L. casei, P. acidilactici*		x	*S. enterica*	oral	x		x	x	x		
23	Lan et al. (2020)	corn, bran, soybean meal, rapeseed meal	*P. pentosaceus*		x	*S. enteritidis*	oral		x	x		x		
24	Wang et al. (2017)	corn, soybean meal	*L. johnsonii*		x	*E. maxima, E. acervulina, C. perfringens*	oral		x			x		x

25	Li et al. (2018)	corn, soybean meal	*L. acidophilus*	x	x	*C. perfringens*	oral		x		x	x	x	
26	Wang et al. (2017)	corn, soybean meal, fish meal	*L. plantarum*		x	*Escherichia coli*	oral		x	x		x	x	
27	Chen et al (2022)	corn, soybean meal	*L. salivarius*		x	*S. pullorum*, Aflatoxin B1	oral, diet			x	x			
28	Calik et al. (2019)	corn, soybean meal	*B. amyloliquefaciens*		x	*E. maxima, E. tenella, E. acervulina*	oral		x	x		x	x	
29	Qiu et al. (2021)	corn, soybean meal	*B. subtilis*	x					x	x	x		x	
30	Cao et al. (2019)	corn, soybean meal	*L. plantarum*		x	*C. perfringens*	oral			x		x		
31	Li et al. (2017)	wheat, soybean meal	*B. subtilis*	x	x	*C. perfringens*	oral		x	x		x	x	
32	Aliakbarpour e al. (2012)	corn, soybean meal	*B. subtilis, L. casei, L. acidophilus, Bi. Thermophilum, E. faecium*	x					x		x		x	x
33	Gao et al. (2022)	corn, soybean meal, fish meal	*L. plantarum*	x					x		x		x	x
34	Hosseini et al. (2019)	corn, soybean meal	*B. subtilis, B. tequilensis*	x						x	x		x	x
35	Konieczka et al (2022)	corn, wheat, soybean meal	*B. licheniformis, B. amyloliquefaciens, B. subtilis*	x	x	*C. perfringens, E. maxima, E. tenella, E. acervulina, E. mitis, E. praecox*	oral	x	x	x	x		x	x
36	Hosseini et al. (2018)	triticale, soybean meal, corn gluten meal	*B. subtilis, B. licheniformis*	x					x		x			
37	Gong et al. (2021)	corn, wheat, soybean meal, fish meal	*L. plantarum, Pa. polymyxa*		x	*C. perfringens*	oral			x	x	x		
38	Zhao et al (2022)	corn, soybean meal	*B. licheniformis*		x	*C. perfringens*	oral		x		x			
39	Liu et al. (2022)	corn, soybean meal, fish meal, rapeseed meal	*C. butyricum*	x					x	x	x	x	x	x

40	Guo et al. (2021)	barley, corn, soybean meal, fish meal	*L. fermentum, B. coagulans*	x	x	*C. perfringens*	oral		x				x		
41	Emami et al. (2019)	corn, soybean meal	*L. acidophilus, L. casei, E. faecium, Bi. Bifidum*		x	*C. perfringens, E. maxima, E. tenella, E. acervulina, E. mivati*	spray		x			x	x		x
42	Mohammed et al. (2021)	corn	*St. faecalis, C. butyricum, B. mesentericus*		x	*S. minnesota*	oral				x		x		
43	Mohsin et al. (2022)	corn, soybean meal	*L. plantarum*		x	*E. tenella*	oral				x	x			
44	Wu et al. (2018)	corn, soybean meal, corn gluten meal	*L. plantarum*		x	*F. graminearum*	diet			x		x	x		
45	Wang et al. (2022)	corn, soybean meal	*B. subtilis*		x	*S. pullorum*	oral				x	x			
46	Deng et al. (2020)	corn, soybean meal, corn gluten meal	*L. acidophilus, L. plantarum*		x	*L. monocytogenes*	oral				x		x		
47	Ateya et al. (2019)	corn, soybean meal, corn gluten meal	*P. acidilactici*		x	*Escherichia coli*	oral			x			x		
48	Zhang et al. (2022)	corn, soybean meal, corn gluten meal	*B. amyloliquefaciens*		x	*C. perfringens*	spray		x			x	x		x
49	Dong et al. (2016)	corn, soybean meal, cottonseed meal	*E. fecalis*	x					x	x		x		x	x
50	Xiao et al. (2022)	corn, soybean meal	*B. methylotrophicus*	x					x	x		x		x	x
51	Azizi et al. (2021)	corn, rice, soybean meal	*L. acidophilus, L. animalis, L. reuteri, L. fermentum, L. gallinarum, S. cerevisiae*		x	*F. graminearum*	diet	x	x	x		x		x	
52	Emami et al. (2020)	corn, soybean meal	*B. licheniformis*	x	x	*C. perfringens, E. maxima, E. tenella, E. acervulina, E. mivati*	spray		x	x		x	x	x	

53	Gharib-Naseri et al. (2020)	wheat, soybean meal, sorghum	*B. amyloliquefaciens*	x	x	*C. perfringens, E. maxima, E. acervulina, E. brunetti*	oral	x		x	x
54	Bodinga et al. (2020)	corn, soybean meal	*B. subtilis*		x	*C. perfringens*	oral	x	x		x

[1] *B, Bacillus; Bi, Bifidobacterium; C, Clostridium; E, Enterococcus; L, Lactobacillus; Pa, Paenibacillus; P, Pediococcus; S, Saccharomyces; St, Streptococcus.*

[2] *C, Clostridium; E, Eimeria; F, Fusarium; L, Listeria; S, Salmonella.*

[3] Main energy and protein feedstuffs of basal diet.

D, duodenum; J, jejunum; I, ileum; C, caeca; BAR, gut barrier-related gene expression; IMU, gut immunity-related gene expression; HIS, histomorphology; GRO, growth performance.

Table S2. Descriptive statistics for predictive and response variables of jejunal and ileal gene expression (fold-change) related to barrier function and immune response in broiler chickens at week 3 and 6 of life without pathogen challenge.

Variable[1,2]	nTreat	Mean	SEM	Min.	Max.	Median
Jejunum, Week 3						
Dietary probiotics (log_{10} CFU/kg)	17	4.8	1.16	0	11.0	6.6
Dietary ME (MJ/kg)	17	12.4	0.06	12.1	12.9	12.4
Dietary CP (%)	17	21.3	0.14	20.5	22.1	21.0
MUC2	10	1.2	0.13	0.9	2.3	1.0
ZO1	11	1.1	0.04	0.9	1.4	1.0
OCLN	13	1.4	0.20	0.9	3.5	1.0
CLDN1	11	1.2	0.10	0.9	1.9	1.0
Jejunum, Week 3						
Dietary probiotics (log_{10} CFU/kg)	22	4.7	0.96	0	11.0	6.8
Dietary ME (MJ/kg)	22	12.4	0.05	12.1	12.9	12.3
Dietary CP (%)	22	21.3	0.11	20.5	22.0	21.0
IL1B	11	1.0	0.04	0.6	1.1	1.0
IFNG	14	1.0	0.04	0.8	1.4	1.0
TLR2	11	1.1	0.12	0.7	2.1	1.0
Jejunum, Week 6						
Dietary probiotics (log_{10} CFU/kg)	24	5.8	0.95	0	11.0	8.7
Dietary ME (MJ/kg)	24	13.0	0.06	12.6	13.4	13.2
Dietary CP (%)	24	19.5	0.15	18.2	20.7	19.3
MUC2	10	1.8	0.37	1.0	4.5	1.3
ZO1	14	1.7	0.33	1.0	5.6	1.2
OCLN	16	1.4	0.18	0.6	3.1	1.0
CLDN1	12	1.4	0.18	0.5	2.6	1.3
Ileum, Week 3						
Dietary probiotics (log_{10} CFU/kg)	13	5.7	1.32	0	11.3	8.0
Dietary ME (MJ/kg)	13	12.3	0.05	12.1	12.6	12.2
Dietary CP (%)	13	21.5	0.18	21.0	22.4	21.0
MUC2	10	2.0	0.64	0.7	7.5	1.0
ZO1	11	1.2	0.19	0.1	2.3	1.0
OCLN	13	1.8	0.62	0.7	9.0	1.0
CLDN1	10	1.3	0.15	0.7	2.2	1.0
Ileum, Week 6						
Dietary probiotics (log_{10} CFU/kg)	19	6.2	0.99	0	10.4	8.5
Dietary ME (MJ/kg)	19	12.9	0.06	12.6	13.3	12.8
Dietary CP (%)	19	19.4	0.18	18.1	20.7	19.2
MUC2	15	1.9	0.39	0.7	5.2	1.0
ZO1	14	2.3	0.63	0.5	8.6	1.0
OCLN	16	1.3	0.17	0.7	3.4	1.1

CLDN1	11	1.5	0.26	0.6	3.1	1.0

n_{Treat}, number of treatments mean; SEM, standard error of means; Min, minimum; Max, maximum; ME, metabolizable energy; CP, crude protein; CFU, colony-forming unit; *MUC2*, mucin-2; *ZO1*, zonula occludens-1; *OCLN*, occludin; *CLDN1*, claudin-1; *IL1B*, interleukin-1beta; *TLR2*, toll-like receptor-2; *IFNG*, interferon-gamma.

[1]Probiotic genera included for these response variables were *Bacillus, Bifidobacterium, Lactobacillus, Clostridium, Enterococcus, Pediococcus, Paenibacillus,* and *Saccharomyces.*

[2]Data were calculated as log_2fold change between probiotic and control treatments and then expressed in fold-change using a logarithmic scale to base 2.

Table S3. Descriptive statistics for predictive and response variables of jejunal and ileal histomorphology (fold-change) in broiler chickens at week 3 and 6 of life without pathogen challenge.

Variable[1,2]	nTreat	Mean	SEM	Min.	Max.	Median
Jejunum, Week 3						
Dietary probiotics (log_{10} CFU/kg)	15	5.0	1.27	0	11.0	6.6
Dietary ME (MJ/kg)	15	12.5	0.07	12.1	12.9	12.5
Dietary CP (%)	15	21.3	0.19	19.9	22.1	21.5
Villus Height	15	1.1	0.04	1.0	1.5	1.0
Crypt Depth	15	1.0	0.04	0.7	1.3	1.0
Villus Height/Crypt Depth	15	1.2	0.08	0.9	1.9	1.0
Jejunum, Week 6						
Dietary probiotics (log_{10} CFU/kg)	19	5.9	1.08	0	11.0	9.0
Dietary ME (MJ/kg)	19	12.9	0.06	12.5	13.2	12.9
Dietary CP (%)	19	19.4	0.16	18.2	20.4	19.2
Villus Height	19	1.1	0.03	0.9	1.3	1.0
Crypt Depth	19	1.1	0.04	0.7	1.5	1.0
Villus Height/Crypt Depth	19	1.0	0.04	0.8	1.5	1.0
Ileum, Week 3						
Dietary probiotics (log_{10} CFU/kg)	11	5.1	1.50	0	11.0	8.5
Dietary ME (MJ/kg)	11	12.4	0.06	12.1	12.7	12.4
Dietary CP (%)	11	21.6	0.16	21.0	22.1	22.0
Villus Height	11	1.0	0.03	0.9	1.3	1.0
Crypt Depth	11	0.9	0.04	0.6	1.1	1.0
Villus Height/Crypt Depth	11	1.1	0.06	0.9	1.7	1.0
Ileum, Week 6						
Dietary probiotics (log_{10} CFU/kg)	17	6.1	1.14	0	11.0	9.0
Dietary ME (MJ/kg)	17	13.0	0.06	12.6	13.2	13.0
Dietary CP (%)	17	19.6	0.15	18.9	20.7	19.2
Villus Height	17	1.1	0.04	0.9	1.5	1.0
Crypt Depth	17	1.0	0.03	0.7	1.2	1.0
Villus Height/Crypt Depth	17	1.1	0.04	0.8	1.4	1.1

nTreat**,** number of treatments means; SEM, standard error of means; Min, minimum; Max, maximum; ME, metabolizable energy; CP, crude protein; CFU, colony-forming unit.

[1]Probiotic genera included for these response variables were *Bacillus, Bifidobacterium, Lactobacillus, Clostridium, Enterococcus,* and *Saccharomyces.*

[2]Data were calculated as log_2fold change between probiotic and control treatments and then expressed in fold-change using a logarithmic scale to base 2.

Table S4. Descriptive statistics for predictors and response variables of growth performance in broiler chickens at starter, finisher, and overall periods without pathogen challenge.

Variable[1]	n_{Treat}	Mean	SEM	Min.	Max.	Median
Starter (1-3 Weeks)						
Dietary probiotics ($\log_{10}$ CFU/kg)	33	5.6	0.80	0	11.0	8.5
Dietary ME (MJ/kg)	33	12.4	0.03	12.1	12.8	12.5
Dietary CP (%)	33	21.5	0.13	21.0	23.0	21.1
ADFI (g)	33	50.2	1.52	35.9	65.3	49.6
ADG (g)	30	33.7	0.82	25.8	44.6	33.7
FCR	33	1.5	0.03	1.1	1.8	1.4
Finisher (4-6 Weeks)						
Dietary probiotics ($\log_{10}$ CFU/kg)	29	5.7	0.86	0	11.0	8.5
Dietary ME (MJ/kg)	29	12.8	0.04	12.5	13.2	12.8
Dietary CP (%)	29	19.5	0.11	18.9	20.7	19.2
ADFI (g)	29	151.8	4.69	105.9	199.7	153.0
ADG (g)	26	77.6	2.98	51.0	107.2	77.2
FCR	29	2.0	0.04	1.5	2.3	1.9
Overall (1-6 Weeks)						
Dietary probiotics ($\log_{10}$ CFU/kg)	32	5.7	0.80	0	11.0	8.5
Dietary ME (MJ/kg)	32	12.7	0.03	12.3	13.0	12.6
Dietary CP (%)	32	20.6	0.11	19.7	21.5	20.3
ADFI (g)	32	100.5	2.79	71.4	132.0	99.5
ADG (g)	26	56.5	1.57	42.1	71.3	56.7
FCR	32	1.7	0.02	1.4	2.0	1.7

n_{Treat}, number of treatments means included; SEM, standard error of means; Min, minimum; Max, maximum; ME, metabolizable energy; CP, crude protein; CFU, colony-forming unit; ADFI, average daily feed intake; ADG, average daily weight gain; FCR, feed conversion ratio.

[1]Probiotic genera included for these response variables were *Bacillus, Bifidobacterium, Lactobacillus, Clostridium, Enterococcus,* and *Saccharomyces.*

Table S5. Descriptive statistics for predictive and response variables of jejunal, ileal, and caecal gene expression (fold-change) related to barrier function in broiler chickens from week 2 to 5 with pathogen challenge.

Variable[1-3]	n_{Treat}	Mean	SEM	Min.	Max.	Median
Jejunum, Week 2						
Dietary probiotics (log_{10} CFU/kg)	18	4.9	1.07	0	10.7	8.2
Days post-infection	18	6.6	0.78	3.0	12.0	6.0
Dietary ME (MJ/kg)	18	12.5	0.05	12.1	12.8	12.6
Dietary CP (%)	18	21.8	0.24	20.0	23.0	21.8
ZO1	14	1.1	0.04	0.7	1.3	1.0
OCLN	16	1.6	0.62	0.6	10.8	1.0
CLDN1	14	1.1	0.17	0.6	3.3	1.0
CLDN3	10	1.5	0.17	1.0	2.3	1.2
Jejunum, Week 3						
Dietary probiotics (log_{10} CFU/kg)	19	5.3	1.07	0	10.7	8.7
Days post-infection	19	7.0	0.59	4.0	12.0	7.0
Dietary ME (MJ/kg)	19	12.5	0.06	12.1	12.9	12.4
Dietary CP (%)	19	20.9	0.12	20.0	22.0	21.0
MUC2	10	1.2	0.19	0.7	2.8	1.0
ZO1	17	1.2	0.08	0.9	2.0	1.0
OCLN	17	1.3	0.14	0.7	3.1	1.0
CLDN1	14	1.6	0.45	0.4	7.3	1.0
Jejunum, Week 4						
Dietary probiotics (log_{10} CFU/kg)	12	5.0	1.28	0	9.2	7.7
Days post-infection	12	12.0	1.69	7.0	21.0	10.0
Dietary ME (MJ/kg)	12	12.6	0.07	12.4	13.0	12.5
Dietary CP (%)	12	20.0	0.28	19.0	21.2	19.8
ZO1	12	1.3	0.10	0.9	2.0	1.1
OCLN	12	1.3	0.20	0.6	2.8	1.0
Jejunum, Week 5						
Dietary probiotics (log_{10} CFU/kg)	13	6.9	1.10	0	9.6	9.0
Days post-infection	13	28.8	2.26	17.0	34.0	34.0
Dietary ME (MJ/kg)	13	12.7	0.03	12.5	12.8	12.8
Dietary CP (%)	13	19.0	0.02	19.0	19.2	19.0
MUC2	13	1.1	0.20	0.2	2.7	1.0
Ileum, Week 3						
Dietary probiotics (log_{10} CFU/kg)	16	5.6	1.12	0	9.7	8.4
Days post-infection	16	14.3	1.62	4.0	20.0	16.0
Dietary ME (MJ/Kg)	16	12.3	0.04	12.1	12.6	12.2
Dietary CP (%)	16	21.4	0.13	21.0	22.4	21.2
ZO1	16	1.3	0.15	0.4	2.7	1.0
OCLN	16	1.3	0.16	0.4	2.7	1.0
CLDN1	11	1.0	0.14	0.5	2.1	1.0

Ileum, Week 4						
Dietary probiotics (log_{10} CFU/kg)	11	5.1	1.50	0	11.3	8.0
Days post-infection	11	11.6	0.85	8.0	14.0	14.0
Dietary ME (MJ/kg)	11	12.8	0.15	12.1	13.2	13.2
Dietary CP (%)	11	20.5	0.17	20.0	21.2	20.0
ZO1	11	1.2	0.11	1.0	2.2	1.1
OCLN	11	1.3	0.18	0.8	2.9	1.0
CLDN1	11	1.3	0.21	0.5	2.7	1.0
Caeca, Week 4						
Dietary probiotics (log_{10} CFU/kg)	10	4.6	1.29	0	8.7	6.5
Days post-infection	10	7.6	0.16	7.0	8.0	8.0
Dietary ME (MJ/Kg)	10	12.7	0.08	12.6	13.2	12.6
Dietary CP (%)	10	19.2	0.13	19.0	20.0	19.0
ZO1	10	2.6	1.05	1.0	11.9	1.5

n_{Treat}, number of treatments means; SEM, standard error of means; Min, minimum; Max, maximum; ME, metabolizable energy; CP, crude protein; CFU, colony-forming unit; *MUC2*, mucin-2; *ZO1*, zonula occludens-1; *OCLN*, occludin; *CLDN1, -3*, claudin-1, -3.
[1]Probiotic genera included for these response variables were *Bacillus*, *Bifidobacterium*, *Lactobacillus*, *Paenibacillus*, *Clostridium*, *Enterococcus*, *Pediococcus*, and *Saccharomyces.*
[2]Pathogens included for these response variables were *E. coli*, *C. perfringens*, *S. enteritidis*, *E. maxima*, *E. tenella*, *E. acervulina*, *E. mivati*, *E. brunetti*, *E. mitis*, *E. praecox*, *F. graminearum*, *S. pullorum*, and Aflatoxin B1.
[3]Data were calculated as log_2fold change between probiotic and control treatments and then expressed in fold-change using a logarithmic scale to base 2.

Table S6. Descriptive statistics for predictive and response variables of jejunal, ileal, and caecal gene expression (fold-change) related to immune response in broiler chickens from week 2 to 4 of life with pathogen challenge.

Variable[1-3]	nTreat	Mean	SEM	Min.	Max.	Median
Jejunum, Week 2						
Dietary probiotics (log_{10} CFU/kg)	14	4.8	1.15	0	8.9	8.2
Days post-infection	14	7.0	0.96	3.0	12.0	6.0
Dietary ME (MJ/kg)	14	12.4	0.05	12.1	12.6	12.5
Dietary CP (%)	14	21.9	0.21	21.0	23.0	21.8
IL1B	10	1.0	0.03	0.7	1.2	1.0
IL10	14	1.2	0.20	0.2	2.9	1.0
IFNG	10	0.9	0.06	0.5	1.0	0.9
Jejunum, Week 3						
Dietary probiotics (log_{10} CFU/kg)	25	5.2	0.95	0	10.7	8.5
Days post-infection	25	8.2	0.85	4.0	20.0	7.0
Dietary ME (MJ/kg)	25	12.4	0.05	12.1	12.9	12.4
Dietary CP (%)	25	21.0	0.13	20.0	22.0	21.0
IL1B	17	0.8	0.07	0.2	1.3	1.0
IL6	12	0.9	0.08	0.4	1.1	1.0
IL10	13	1.7	0.33	0.5	4.6	1.0
IFNG	18	0.9	0.07	0.4	1.5	1.0
TNFA	10	0.9	0.06	0.6	1.2	1.0
Jejunum, Week 4						
Dietary probiotics (log_{10} CFU/kg)	14	5.1	1.23	0	10.0	7.7
Days post-infection	14	11.8	1.04	7.0	17.0	13.5
Dietary ME (MJ/kg)	14	12.7	0.09	12.4	13.2	12.6
Dietary CP (%)	14	19.7	0.21	19.0	21.0	19.5
IL1B	10	1.4	0.14	1.0	2.2	1.2
IFNG	14	1.2	0.13	0.6	2.4	1.0
Ileum, Week 2						
Dietary probiotics (log_{10} CFU/kg)	12	4.6	1.39	0	9.7	4.1
Days post-infection	12	3.5	0.77	1.0	7.0	2.5
Dietary ME (MJ/kg)	12	12.6	0.12	12.1	13.2	12.8
Dietary CP (%)	12	21.6	0.18	20.9	22.1	21.6
IFNG	10	1.0	0.16	0.4	2.2	1.0
TLR4	10	0.9	0.10	0.5	1.6	1.0
Ileum, Week 3						
Dietary probiotics (log_{10} CFU/kg)	12	5.1	1.32	0	9.7	8.0
Days post-infection	12	13.7	2.27	4.0	20.0	20.0
Dietary ME (MJ/kg)	12	12.3	0.05	12.1	12.5	12.2
Dietary CP (%)	12	21.4	0.17	21.0	22.4	21.1
IL10	10	1.1	0.11	0.7	2.0	1.0
IFNG	12	0.9	0.05	0.6	1.1	0.9

Ileum, Week 4						
Dietary probiotics (log_{10} CFU/kg)	10	5.9	1.63	0	11.3	9.0
Days post-infection	10	17.3	2.60	8.0	27.0	17.0
Dietary ME (MJ/kg)	10	12.4	0.11	12.1	12.8	12.1
Dietary CP (%)	10	19.8	0.41	19.0	22.1	19.0
TNFA	10	0.8	0.09	0.2	1.0	1.0
Caeca, Week 2						
Dietary probiotics (log_{10} CFU/kg)	22	5.5	1.04	0	12.4	8.0
Days post-infection	22	3.4	0.60	0.2	7.0	3.0
Dietary ME (MJ/kg)	22	12.0	0.10	11.4	12.8	12.1
Dietary CP (%)	22	20.8	0.25	19.2	23.0	21.0
IL6	18	0.9	0.07	0.1	1.5	1.0
IL8	10	0.9	0.19	0.1	2.1	1.0
IL10	10	2.4	0.42	1.0	4.7	2.5

n_{Treat}, number of treatments means; SEM, standard error of means; Min, minimum; Max, maximum; ME, metabolizable energy; CP, crude protein; CFU, colony-forming unit; *IL6, -8, -10, -1B*, interleukin-6, -8, -10, -1B; *TLR4*, toll-like receptor -4; *IFNG*, interferon-gamma; *TNFA*, tumor necrosis factor-alpha.

[1]Probiotic genera included for these response variables were *Bacillus*, *Bifidobacterium, Lactobacillus*, *Paenibacillus*, *Clostridium*, *Enterococcus, Pediococcus*, *Streptococcus,* and *Saccharomyces.*

[2]Pathogens included for these response variables were *E. coli, C. perfringens, S. enteritidis, E. maxima, E. tenella, E. acervulina, E. mivati, E. brunetti, F. graminearum, S. minnesota,* and *L. monocytogenes.*

[3]Data were calculated as log_2fold change between probiotic and control treatments and then expressed in fold-change using a logarithmic scale to base 2.

Table S7. Descriptive statistics for predictive and response variables of duodenal, jejunal and ileal histomorphology (fold-change) in broiler chickens at week 2, 3 and 5 of life with pathogen challenge.

Variable[1-3]	nTreat	Mean	SEM	Min.	Max.	Median
Duodenum, Week 5						
Dietary probiotics (log_{10} CFU/kg)	15	7.2	0.97	0	9.4	9.0
Days post-infection	15	32.0	1.07	24.0	34.0	34.0
Dietary ME (MJ/kg)	15	12.8	0.02	12.8	13.0	12.8
Dietary CP (%)	15	19.2	0.11	19.0	20.0	19.0
Villus Height	15	1.1	0.02	1.0	1.3	1.1
Crypt Depth	15	1.1	0.03	0.8	1.2	1.0
Villus Height/Crypt Depth	15	1.0	0.03	0.9	1.3	1.0
Jejunum, Week 2						
Dietary probiotics (log_{10} CFU/kg)	11	5.2	1.50	0	10.7	8.7
Days post-infection	11	5.2	0.48	3.0	7.0	5.0
Dietary ME (MJ/kg)	11	12.4	0.09	12.1	12.8	12.4
Dietary CP (%)	11	21.2	0.25	20.0	22.1	21.0
Villus Height	11	1.0	0.04	0.9	1.4	1.0
Crypt Depth	11	0.9	0.03	0.7	1.0	1.0
Villus Height/Crypt Depth	11	1.1	0.08	1.0	1.8	1.0
Jejunum, Week 3						
Dietary probiotics (log_{10} CFU/kg)	17	5.3	1.12	0	10.7	8.2
Days post-infection	17	9.8	1.34	4.0	20.0	7.0
Dietary ME (MJ/kg)	17	12.3	0.03	12.1	12.5	12.3
Dietary CP (%)	17	21.1	0.12	20.0	22.0	21.0
Villus Height	17	1.1	0.04	0.9	1.5	1.0
Crypt Depth	17	1.0	0.03	0.7	1.1	1.0
Villus Height/Crypt Depth	17	1.2	0.07	1.0	2.2	1.1
Jejunum, Week 5						
Dietary probiotics (log_{10} CFU/kg)	17	6.9	0.96	0	9.4	9.0
Days post-infection	17	30.6	1.35	20.0	34.0	34.0
Dietary ME (MJ/kg)	17	12.8	0.03	12.6	13.0	12.8
Dietary CP (%)	17	19.2	0.09	19.0	20.0	19.0
Villus Height	17	1.0	0.02	0.9	1.2	1.0
Crypt Depth	17	1.0	0.03	0.7	1.1	1.0
Villus Height/Crypt Depth	17	1.1	0.04	0.8	1.4	1.1
Ileum, Week 3						
Dietary probiotics (log_{10} CFU/kg)	13	5.5	1.26	0	9.7	8.5
Days post-infection	13	10.3	1.73	4.0	20.0	7.0
Dietary ME (MJ/kg)	13	12.3	0.04	12.1	12.5	12.3
Dietary CP (%)	13	21.2	0.10	21.0	22.0	21.1

Villus Height	13	1.0	0.02	1.0	1.2	1.0
Crypt Depth	13	1.0	0.04	0.9	1.4	1.0
Villus Height/Crypt Depth	13	1.0	0.02	0.9	1.2	1.0
Ileum, Week 5						
Dietary probiotics (log_{10} CFU/kg)	15	7.2	0.97	0	9.4	9.0
Days post-infection	15	32.0	1.07	24.0	34.0	34.0
Dietary ME (MJ/kg)	15	12.8	0.02	12.8	13.0	12.8
Dietary CP (%)	15	19.2	0.11	19.0	20.0	19.0
Villus Height	15	1.0	0.03	0.8	1.2	1.0
Crypt Depth	15	1.1	0.03	0.9	1.3	1.1
Villus Height/Crypt Depth	15	0.9	0.03	0.7	1.1	1.0

n_{Treat}, number of treatments means; SEM, standard error of means; Min, minimum; Max, maximum; ME, metabolizable energy; CP, crude protein; CFU, colony-forming unit.

[1]Probiotic genera included for these response variables were *Bacillus*, *Lactobacillus*, *Enterococcus,* and *Saccharomyces.*

[2]Pathogens included for these response variables were *E. coli, C. perfringens, S. enteritidis, E. maxima, E. tenella, E. acervulina, E. mitis, E. praecox,* and *F. graminearum.*

[3]Data were calculated as log_2fold change between probiotic and control treatments and then expressed in fold-change using a logarithmic scale to base 2.

Table S8. Descriptive statistics for predictors and response variables of growth performance in broiler chickens at starter, finisher, and overall periods with pathogen challenge.

**Variable**[1,2]	**nTreat**	**Mean**	**SEM**	**Min.**	**Max.**	**Median**
Starter (1-3 Weeks)						
Dietary probiotics (log_{10} CFU/kg)	22	5.5	1.00	0	9.7	9.0
Days post-infection	22	10.8	1.00	5.0	20.0	10.0
Dietary ME (MJ/kg)	22	12.4	0.04	12.1	12.7	12.4
Dietary CP (%)	22	21.1	0.05	21.0	21.8	21.0
ADFI (g)	22	52.6	1.57	40.6	63.3	52.3
ADG (g)	22	36.2	1.46	28.4	50.2	33.2
FCR	22	1.5	0.04	1.1	1.9	1.4
Finisher (4-6 Weeks)						
Dietary probiotics (log_{10} CFU/kg)	13	5.0	1.33	0	9.6	9.0
Days post-infection	13	33.2	1.50	27.0	41.0	33.0
Dietary ME (MJ/kg)	13	12.7	0.05	12.5	13.0	12.8
Dietary CP (%)	13	19.3	0.10	19.0	19.8	19.0
ADFI (g)	13	149.8	11.04	108.2	219.4	142.7
ADG (g)	13	72.1	7.54	31.7	114.3	80.7
FCR	13	2.0	0.09	1.7	2.8	1.9
Overall (1-6 Weeks)						
Dietary probiotics (log_{10} CFU/kg)	17	4.8	1.13	0	9.6	8.1
Days post-infection	17	34.9	1.38	27.0	41.0	36.0
Dietary ME (MJ/kg)	17	12.7	0.06	12.4	13.0	12.5
Dietary CP (%)	17	20.3	0.08	19.9	20.8	20.1
ADFI (g)	17	95.6	2.78	75.6	119.5	93.8
ADG (g)	17	57.0	3.26	31.7	75.7	55.2
FCR	17	1.7	0.06	1.4	2.3	1.7

nTreat, number of treatments means; SEM, standard error of means; Min, minimum; Max, maximum; ME, metabolizable energy; CP, crude protein; CFU, colony-forming unit; ADFI, average daily feed intake; ADG, average daily weight gain; FCR, feed conversion ratio.

[1]Probiotic genera included for these response variables were *Bacillus, Bifidobacterium, Lactobacillus, Enterococcus,* and *Saccharomyces.*

[2]Pathogens included for these response variables were *E. coli, C. perfringens, S. enteritidis, E. maxima, E. tenella, E. acervulina, E. mivati, E. mitis,* and *E. praecox.*

References

Aliakbarpour, H.R.; Chamani, M.; Rahimi, G.; Sadeghi, A.A.; Qujeq, D. The *Bacillus subtilis* and lactic acid bacteria probiotics influences intestinal mucin gene expression, histomorphology and growth performance in broilers. *Asian-Australasian J. Anim. Sci.* **2012**, *25*, 1285–1293, doi:10.5713/ajas.2012.12110.

Ateya, A.I.; Arafat, N.; Saleh, R.M.; Ghanem, H.M.; Naguib, D.; Radwan, H.A.; Elseady, Y.Y. Intestinal gene expressions in broiler chickens infected with *Escherichia coli* and dietary supplemented with probiotic, acidifier and synbiotic. *Vet. Res. Commun.* **2019**, *43*, 131–142, doi:10.1007/s11259-019-09753-z.

Azizi, T.; Daneshyar, M.; Allymehr, M.; Tukmechi, A.; Khalilvandi Behroozyar, H.; Shalizar Jalali, A. Combination of *Lactobacillus* species and yeast ameliorates adverse effect of deoxynivalenol contaminated diet on immune system, gut morphology and jejunal gene expression in broiler chickens. *Ital. J. Anim. Sci.* **2021**, *20*, 59–70, doi:10.1080/1828051X.2020.1865845.

Bai, S.P.; Wu, A.M.; Ding, X.M.; Lei, Y.; Bai, J.; Zhang, K.Y.; Chio, J.S. Effects of probiotic-supplemented diets on growth performance and intestinal immune characteristics of broiler chickens. *Poult. Sci.* **2013**, *92*, 663–670, doi:https://doi.org/10.3382/ps.2012-02813.

Bilal, M.; Si, W.; Barbe, F.; Chevaux, E.; Sienkiewicz, O.; Zhao, X. Effects of novel probiotic strains of *Bacillus pumilus* and *Bacillus subtilis* on production, gut health, and immunity of broiler chickens raised under suboptimal conditions. *Poult. Sci.* **2021**, *100*, 100871, doi:10.1016/j.psj.2020.11.048.

Bodinga, B.M.; Hayat, K.; Liu, X.; Zhou, J.; Yang, X.; Ismaila, A.; Soomro, R.N.; Ren, Z.; Zhang, W.; Yang, X.J. Effects of *Bacillus Subtilis* DSM 32315 on immunity, nutrient transporters and functional diversity of cecal microbiome of broiler chickens in necrotic enteritis challenge. *J. World's Poult. Res.* **2020**, *10*, 527–544, doi:10.36380/jwpr.2020.61.

Calik, A.; Omara, I.I.; White, M.B.; Li, W.; Dalloul, R.A. Effects of dietary direct fed microbial supplementation on performance, intestinal morphology and immune response of broiler chickens challenged with coccidiosis. *Front. Vet. Sci.* **2019**, *6*, 463, doi:10.3389/fvets.2019.00463.

Cao, L.; Wu, X.H.; Bai, Y.L.; Wu, X.Y.; Gu, S.B. Anti-inflammatory and antioxidant activities of probiotic powder containing *Lactobacillus plantarum* 1.2567 in necrotic enteritis model of broiler chickens. *Livest. Sci.* **2019**, *223*, 157–163, doi:10.1016/j.livsci.2019.03.009.

Chang, C.H.; Teng, P.Y.; Lee, T.T.; Yu, B. Effects of multi-strain probiotic supplementation on intestinal microbiota, tight junctions, and inflammation in young broiler chickens challenged with *Salmonella enterica* subsp. enterica. *Asian-Australasian J. Anim. Sci.* **2020**, *33*, 1797–1808, doi:10.5713/ajas.19.0427.

Chaudhari, A.A.; Lee, Y.; Lillehoj, H.S. Beneficial effects of dietary supplementation of *Bacillus* strains on growth performance and gut health in chickens with mixed coccidiosis infection. *Vet. Parasitol.* **2020**, *277*, 109009, doi:https://doi.org/10.1016/j.vetpar.2019.109009.

Chen, X.; Ishfaq, M.; Wang, J. Effects of *Lactobacillus salivarius* supplementation on the growth performance, liver function, meat quality, immune responses and *Salmonella Pullorum* infection resistance of broilers challenged with Aflatoxin B1. *Poult. Sci.* **2022**, *101*, doi:10.1016/j.psj.2021.101651.

Deng, Q.; Shi, H.; Luo, Y.; Zhao, H.; Liu, N. Effect of dietary *Lactobacilli* mixture on *Listeria monocytogenes* infection and virulence property in broilers. *Poult. Sci.* **2020**, *99*, 3655–3662, doi:10.1016/j.psj.2020.03.058.

Dong, Z.L.; Wang, Y.W.; Song, D.; Hou, Y.J.; Wang, W.W.; Qi, W.T.; Yun, T.T.; Li, A.K. The effects of dietary supplementation of pre-microencapsulated *Enterococcus fecalis* and the extract of Camellia oleifera seed on growth performance, intestinal morphology, and intestinal mucosal immune functions in broiler chickens. *Anim. Feed Sci. Technol.* **2016**, *212*, 42–51, doi:https://doi.org/10.1016/j.anifeedsci.2015.11.014.

Emami, N.K.; Calik, A.; White, M.B.; Kimminau, E.A.; Dalloul, R.A. Effect of probiotics and multi-component feed additives on microbiota, gut barrier and immune responses in broiler chickens during subclinical necrotic enteritis. *Front. Vet. Sci.* **2020**, *7*, 972.

Emami, N.K.; Calik, A.; White, M.B.; Young, M.; Dalloul, R.A. Necrotic enteritis in broiler chickens: the role of tight junctions and

mucosal immune responses in alleviating the effect of the disease. *Microorganisms* **2019**, *7*, 231, doi:10.3390/microorganisms7080231.

Gao, J.; Wang, R.; Liu, J.; Wang, W.; Chen, Y.; Cai, W. Effects of novel microecologics combined with traditional Chinese medicine and probiotics on growth performance and health of broilers. *Poult. Sci.* **2022**, *101*, 101412, doi:10.1016/j.psj.2021.101412.

Gharib-Naseri, K.; de Paula Dorigam, J.C.; Doranalli, K.; Kheravii, S.; Swick, R.A.; Choct, M.; Wu, S.B. Modulations of genes related to gut integrity, apoptosis, and immunity underlie the beneficial effects of *Bacillus amyloliquefaciens* CECT 5940 in broilers fed diets with different protein levels in a necrotic enteritis challenge model. *J. Anim. Sci. Biotechnol.* **2020**, *11*, 1–13, doi:10.1186/s40104-020-00508-4.

Gong, L.; Wang, B.; Zhou, Y.; Tang, L.; Zeng, Z.; Zhang, H.; Li, W. Protective effects of *Lactobacillus plantarum* 16 and *Paenibacillus polymyxa* 10 against *Clostridium perfringens* infection in broilers. *Front. Immunol.* **2021**, *11*, 1–12, doi:10.3389/fimmu.2020.628374.

Guo, S.; Xi, Y.; Xia, Y.; Wu, T.; Zhao, D.; Zhang, Z.; Ding, B. Dietary *Lactobacillus fermentum* and *Bacillus coagulans* supplementation modulates intestinal immunity and microbiota of broiler chickens challenged by *Clostridium perfringens*. *Front. Vet. Sci.* **2021**, *8*, doi:10.3389/fvets.2021.680742.

He, T.; Long, S.; Mahfuz, S.; Wu, D.; Wang, X.; Wei, X.; Piao, X. Effects of probiotics as antibiotics substitutes on growth performance, serum biochemical parameters, intestinal morphology, and barrier function of broilers. *Animals* **2019**, *9*, doi:10.3390/ani9110985.

Hosseini, N.G.; Modarressi, M.H.; Mousavi, S.N.; Ebrahimi, M.T. Effects of indigenous spore-forming probiotic as feed supplement on performance and safety in broilers. *J. Hell. Vet. Med. Soc.* **2019**, *70*, 1841–1850, doi:10.12681/jhvms.22234.

Hosseini, S.M.; Chamani, M.; Mousavi, S.N.; Hosseini, S.A.; Sadeghi, A.A. Growth performance, mucin2 gene expression, morphology of small intestine and intestinal lactobacillus population of broiler chicks fed with triticale-based diets: Effects of dietary physical form and dietary inclusion of enzyme and probiotic. *Kafkas Univ. Vet. Fak. Derg.* **2018**, *24*, 203–210, doi:10.9775/kvfd.2017.18629.

Huang, L.; Luo, L.; Zhang, Y.; Wang, Z.; Xia, Z. Effects of the dietary probiotic, *Enterococcus faecium* NCIMB11181, on the intestinal barrier and system immune status in *Escherichia coli* O78-challenged broiler chickens. *Probiotics Antimicrob. Proteins* **2019**, *11*, 946–956, doi:10.1007/s12602-018-9434-7.

Kan, L.; Guo, F.; Liu, Y.; Pham, V.H.; Guo, Y.; Wang, Z. Probiotics *Bacillus licheniformis* improves intestinal health of subclinical necrotic enteritis-challenged broilers. *Front. Microbiol.* **2021**, *12*, 623739, doi:10.3389/fmicb.2021.623739.

Konieczka, P.; Sandvang, D.; Kinsner, M.; Szkopek, D.; Szyryńska, N.; Jankowski, J. *Bacillus*-based probiotics affect gut barrier integrity in different ways in chickens subjected to optimal or challenge conditions. *Vet. Microbiol.* **2022**, *265*, doi:10.1016/j.vetmic.2021.109323.

Lan, D.; Xun, X.Y.; Hu, Y.D.; Li, N.Z.; Yang, C.W.; Jiang, X.S.; Liu, Y.P. Research on the effect of *Pediococcus pentosaceus* on *Salmonella enteritidis* -infected chicken. *Biomed Res. Int.* **2020**, *2020*, doi:10.1155/2020/6416451.

Li, Y.; Zhang, H.; Chen, Y.P.; Yang, M.X.; Zhang, L.L.; Lu, Z.X.; Zhou, Y.M.; Wang, T. *Bacillus amyloliquefaciens* supplementation alleviates immunological stress and intestinal damage in lipopolysaccharide-challenged broilers. *Anim. Feed Sci. Technol.* **2015**, *208*, 119–131, doi:https://doi.org/10.1016/j.anifeedsci.2015.07.001.

Li, Z.; Wang, W.; Lv, Z.; Liu, D.; Guo, Y. *Bacillus subtilis* and yeast cell wall improve the intestinal health of broilers challenged by *Clostridium perfringens*. *Br. Poult. Sci.* **2017**, *58*, 635–643, doi:10.1080/00071668.2017.1370697.

Li, Z.; Wang, W.; Liu, D.; Guo, Y. Effects of *Lactobacillus acidophilus* on the growth performance and intestinal health of broilers challenged with *Clostridium perfringens*. *J. Anim. Sci. Biotechnol.* **2018**, *9*, 1–10, doi:10.1186/s40104-018-0243-3.

Liu, L.; Ling, H.; Zhang, W.; Zhou, Y.; Li, Y.; Peng, N.; Zhao, S. Functional comparison of *Clostridium butyricum* and sodium butyrate supplementation on growth, intestinal health, and the anti-inflammatory response of broilers. *Front. Microbiol.* **2022**, *13*, 1–11, doi:10.3389/fmicb.2022.914212.

Liu, Y.; Zhang, S.; Luo, Z.; Liu, D. Supplemental *Bacillus subtilis* PB6 improves growth performance and gut health in broilers

challenged with *Clostridium perfringens*. *J. Immunol. Res.* **2021**, *2021*, 2549541, doi:10.1155/2021/2549541.

Memon, F.U.; Yang, Y.; Leghari, I.H.; Lv, F.; Soliman, A.M.; Zhang, W.; Si, H. Transcriptome analysis revealed ameliorative effects of *Bacillus* based probiotic on immunity, gut barrier system, and metabolism of chicken under an experimentally induced *Eimeria tenella* infection. *Genes (Basel).* **2021**, *12*, doi:10.3390/genes12040536.

Mohammed, E.S.I.; Radey, R. Immunomodulation of antimicrobial peptides expression in the gastrointestinal tract by probiotics in response to stimulation by *Salmonella minnesota* Lipopolysaccharides. *Probiotics Antimicrob. Proteins* **2021**, *13*, 1157–1172, doi:10.1007/s12602-021-09746-y.

Mohsin, M.; Zhang, Z.; Yin, G. Effect of probiotics on the performance and intestinal health of broiler chickens infected with *Eimeria tenella. Vaccines* **2022**, *10*, 1–14, doi:10.3390/vaccines10010097.

Mountzouris, K.C.; Palamidi, I.; Paraskeuas, V.; Griela, E.; Fegeros, K. Dietary probiotic form modulates broiler gut microbiota indices and expression of gut barrier genes including essential components for gut homeostasis. *J. Anim. Physiol. Anim. Nutr. (Berl).* **2019**, *103*, 1143–1159, doi:https://doi.org/10.1111/jpn.13112.

Musa, B.B.; Duan, Y.; Khawar, H.; Sun, Q.; Ren, Z.; Elsiddig Mohamed, M.A.; Abbasi, I.H.R.; Yang, X. *Bacillus subtilis* B21 and *Bacillus licheniformis* B26 improve intestinal health and performance of broiler chickens with *Clostridium perfringens* -induced necrotic enteritis. *J. Anim. Physiol. Anim. Nutr. (Berl).* **2019**, *103*, 1039–1049, doi:10.1111/jpn.13082.

Qiu, K.; Li, C.L.; Wang, J.; Qi, G.H.; Gao, J.; Zhang, H.J.; Wu, S.G. Effects of dietary supplementation with *Bacillus subtilis*, as an alternative to antibiotics, on growth performance, serum immunity, and intestinal health in broiler chickens. *Front. Nutr.* **2021**, *8*, 1–13, doi:10.3389/fnut.2021.786878.

Wang, H.; Ni, X.; Qing, X.; Liu, L.; Lai, J.; Khalique, A.; Li, G.; Pan, K.; Jing, B.; Zeng, D. Probiotic enhanced intestinal immunity in broilers against subclinical necrotic enteritis. *Front. Immunol.* **2017**, *8*, 1–14, doi:10.3389/fimmu.2017.01592.

Wang, J.; Ishfaq, M.; Miao, Y.; Liu, Z.; Hao, M.; Wang, C.; Wang, J.; Chen, X. Dietary administration of *Bacillus subtilis* KC1 improves growth performance, immune response, heat stress tolerance, and disease resistance of broiler chickens. *Poult. Sci.* **2022**, *101*, 1–13, doi:10.1016/j.psj.2021.101693.

Wang, S.; Peng, Q.; Jia, H.M.; Zeng, X.F.; Zhu, J.L.; Hou, C.L.; Liu, X.T.; Yang, F.J.; Qiao, S.Y. Prevention of *Escherichia coli* infection in broiler chickens with *Lactobacillus plantarum* B1. *Poult. Sci.* **2017**, *96*, 2576–2586, doi:10.3382/ps/pex061.

Wang, W.; Li, Z.; Han, Q.; Guo, Y.; Zhang, B.; D'inca, R. Dietary live yeast and mannan-oligosaccharide supplementation attenuate intestinal inflammation and barrier dysfunction induced by *Escherichia coli* in broilers. *Br. J. Nutr.* **2016**, *116*, 1878–1888, doi:DOI: 10.1017/S0007114516004116.

Wang, Y.; Xu, Y.; Xu, S.; Yang, J.; Wang, K.; Zhan, X. *Bacillus subtilis* DSM29784 alleviates negative effects on growth performance in broilers by improving the intestinal health under necrotic enteritis challenge. *Front. Microbiol.* **2021**, *12*, 2470.

Wu, S.; Liu, Y.; Duan, Y.; Wang, F.; Guo, F.; Yan, F.; Yang, X.; Yang, X. Intestinal toxicity of deoxynivalenol is limited by supplementation with *Lactobacillus plantarum* JM113 and consequentially altered gut microbiota in broiler chickens. *J. Anim. Sci. Biotechnol.* **2018**, *9*, 74, doi:10.1186/s40104-018-0286-5.

Wu, Y.; Wang, B.; Zeng, Z.; Liu, R.; Tang, L.; Gong, L.; Li, W. Effects of probiotics *Lactobacillus plantarum* 16 and *Paenibacillus polymyxa* 10 on intestinal barrier function, antioxidative capacity, apoptosis, immune response, and biochemical parameters in broilers. *Poult. Sci.* **2019**, *98*, 5028–5039, doi:https://doi.org/10.3382/ps/pez226.

Wu, Y.; Shao, Y.; Song, B.; Zhen, W.; Wang, Z.; Guo, Y.; Shahid, M.S.; Nie, W. Effects of *Bacillus coagulans* supplementation on the growth performance and gut health of broiler chickens with *Clostridium perfringens*-induced necrotic enteritis. *J. Anim. Sci. Biotechnol.* **2018**, *9*, 1–14, doi:10.1186/s40104-017-0220-2.

Wu, Y.; Zhen, W.; Geng, Y.; Wang, Z.; Guo, Y. Pretreatment with probiotic *Enterococcus faecium* NCIMB 11181 ameliorates necrotic enteritis-induced intestinal barrier injury in broiler chickens. *Sci. Rep.* **2019**, *9*, 10256, doi:10.1038/s41598-019-46578-x.

Wu, Z.; Yang, K.; Zhang, A.; Chang, W.; Zheng, A.; Chen, Z.; Cai, H.; Liu, G. Effects of *Lactobacillus acidophilus* on the growth

performance, immune response, and intestinal barrier function of broiler chickens challenged with *Escherichia coli* O157. *Poult. Sci.* **2021**, *100*, 101323, doi:10.1016/j.psj.2021.101323.

Xiao, D.; Wang, Z.; Dai, X.; Hu, Y.; Zhong, M.; Xiong, L.; Jiang, C.; Khalique, A.; Ni, X.; Zeng, D.; et al. Effects of *Bacillus methylotrophicus* SY200 supplementation on growth performance, antioxidant status, intestinal morphology, and immune function in broiler chickens. *Probiotics Antimicrob. Proteins* **2022**, doi:10.1007/s12602-022-09924-6.

Xu, X.; Yang, S.; Olajide, J.S.; Qu, Z.; Gong, Z.; Wang, J.; Zhang, Y.; Wang, H.; Xiong, L.; Zhang, K.; et al. *Clostridium butyricum* supplement can ameliorate the intestinal barrier roles in broiler chickens experimentally infected with *Clostridium perfringens*. *Front. Physiol.* **2021**, *12*, 1572.

Yang, Y.; Memon, F.U.; Hao, K.; Jiang, M.; Guo, L.; Liu, T.; Lv, F.; Zhang, W.; Zhang, Y.; Si, H. The combined use of *Bacillus subtilis*-based probiotic and anticoccidial herb had a better anti-*Eimeria tenella* efficiency. *J. Appl. Poult. Res.* **2021**, *30*, doi:10.1016/j.japr.2021.100181.

Zhang, B.; Zhang, H.; Yu, Y.; Zhang, R.; Wu, Y.; Yue, M.; Yang, C. Effects of *Bacillus coagulans* on growth performance, antioxidant capacity, immunity function, and gut health in broilers. *Poult. Sci.* **2021**, *100*, 101168, doi:10.1016/j.psj.2021.101168.

Zhang, H.; Zhou, Y.; Xu, H.; Liang, C.; Zhai, Z. *Bacillus amyloliquefaciens* BLCC1-0238 alone or in combination with mannan-oligosaccharides alleviates subclinical necrotic enteritis in broilers. *Probiotics Antimicrob. Proteins* **2022**, *14*, 158–168, doi:10.1007/s12602-021-09853-w.

Zhao, Y.; Zeng, Y.; Zeng, D.; Wang, H.; Sun, N.; Xin, J.; Zhou, M.; Yang, H.; Lei, L.; Ling, H.; et al. Dietary probiotic supplementation suppresses subclinical necrotic enteritis in broiler chickens in a microbiota-dependent manner. *Front. Immunol.* **2022**, *13*, 1–18, doi:10.3389/fimmu.2022.855426.

Zhen, W.; Shao, Y.; Gong, X.; Wu, Y.; Geng, Y.; Wang, Z.; Guo, Y. Effect of dietary *Bacillus coagulans* supplementation on growth performance and immune responses of broiler chickens challenged by *Salmonella enteritidis*. *Poult. Sci.* **2018**, *97*, 2654–2666, doi:https://doi.org/10.3382/ps/pey119.

Disclaimer/Publisher's Note: The statements, opinions and data contained in all publications are solely those of the individual author(s) and contributor(s) and not of MDPI and/or the editor(s). MDPI and/or the editor(s) disclaim responsibility for any injury to people or property resulting from any ideas, methods, instructions or products referred to in the content.
